# Prevalence of climate anxiety and its association with mental health: national cross-sectional study in Iraq

**DOI:** 10.1192/bji.2025.10068

**Published:** 2026-08

**Authors:** Darya Rostam Ahmed, Karwan Kakabra Kakamad, Rania H. Al-Taie, Jaafar O. Ahmed, Reinhard Heun

**Affiliations:** 1 Department of Clinical Psychology, Faculty of Science and Health, Koya University, Koya, Kurdistan Region, Iraq.; 2 Department of Psychology, Counseling and Mental Health, Faculty of Arts, Soran University, Soran, Kurdistan Region, Iraq; 3 College of Medicine, Mustansiryia University, Baghdad, Iraq; 4 Medical School, University of Bonn, Bonn, Germany

**Keywords:** Climate anxiety, eco-anxiety, environmental degradation, mental health, Iraq

## Abstract

**Background:**

Iraq is among the countries most vulnerable to climate change, and it is faced with extreme heat, drought and environmental degradation.

**Aims:**

To examine the prevalence of climate anxiety and its association with depression and generalised anxiety disorder in the Iraqi population.

**Method:**

A cross-sectional survey recruited 1019 adult participants (47.8% males, 52.2% females). Most participants were aged 18–41 years (*n* = 854, 83.8%); 16.2% (*n* = 165) were aged 42–72 years. Regionally, 75.6% (*n* = 770) were from the Kurdistan Region and 24.4% (*n* = 249) from provinces in central and southern Iraq. The study used the Climate Change Anxiety Scale (CCAS), Patient Health Questionnaire-9 (PHQ-9) and Generalised Anxiety Disorder-7 (GAD-7). Statistical analyses, included descriptive analysis, analysis of variance (ANOVA), *t*-tests, Pearson’s correlations and regression models, examined variations in climate anxiety by demographics and associations with depression and anxiety.

**Results:**

Overall, 71.4% of participants reported severe climate anxiety, with a higher prevalence in the Kurdistan Region (73.2%) compared with central and southern Iraq (65.9%). Five provinces were found to have significantly higher levels of climate anxiety: Ninawa, Basrah, Najaf, Duhok and Erbil. Age was a significant predictor, and older participants (42–72 years) reported higher levels than younger participants (*P* = 0.008). A positive correlation was observed between climate anxiety and both depression (*r* = 0.382, *P* < 0.001) and generalised anxiety (*r* = 0.361, *P* < 0.001). Simple linear regression revealed that climate anxiety was significantly associated with both depression (*β* = 0.25, *P* < 0.001) and generalised anxiety (*β* = 0.214, *P* < 0.01), accounting for the 14.6 and 13% variance, respectively.

**Conclusions:**

Climate anxiety is prevalent in Iraq and significantly associated with mental health problems. The findings endorse the need for integration of mental health into Iraq’s national climate adaptation and public health policies.

Climate change refers to long-term changes in weather patterns and temperatures. Human activities have been the main cause of climate change since the 1800s, mainly due to the combustion of fossil fuels such as coal, oil and gas.^1^ On the basis of its causes and consequences, climate change can be classified into various categories. The two main categories of climate change are man-made climate changes caused by greenhouse gas emissions and industrial activity,^2^ and natural changes driven by long-term processes such as solar variability and volcanic activity.^3^ Man-made changes tend to occur more rapidly, often within decades, whereas natural processes usually unfold over centuries or longer.^3^ In addition, certain localised forces such as urbanisation or land-use change can create region-specific climate effects,^4^ but these are not considered a separate category. The World Health Organization (WHO) estimates that 3.6 billion people currently live in regions that are particularly vulnerable to climate change and estimates that approximately 250 000 additional deaths per year will be the result of climate change between 2030 and 2050.^4^ An increased level of psychological, mental or physical distress in response to hazardous changes in the climate system is known as climate anxiety. Symptoms of climate anxiety include vulnerability, irritability, panic attacks, appetite loss and insomnia.^5^ Eco-anxiety refers to a chronic fear of environmental doom that can range from mild worry to severe psychological distress, including symptoms such as anxiety, depression, post-traumatic stress disorder (PTSD) and suicidal thoughts.^7^ At-risk groups, such as children, elderly people, people with mental illness, people with mobility disabilities and those who are chronically ill, must be considered when assessing the effects of climate anxiety.^8^ Moreover, climate change has a disproportionate impact on people in lower socioeconomic categories, because of differences in infrastructure and health resources.^5^ Furthermore, research has revealed that the prevalence of PTSD is 26% and depression is 21.7% in populations affected by catastrophic weather events such as hurricanes and floods.^9^ According to a population-based study in Taiwan, for every 1°C increase in temperature, there is a 7% increase in major depressive disorder diagnoses, particularly among older people.^10^

## Climate change in Iraq

Iraq is a country located in the Middle East, and the Kurdistan Region of Iraq (KRI) is an autonomous region in northern Iraq. The Kurdistan Region consists of four governorates: Erbil, Sulaymaniyah, Duhok and Halabja. It is a constitutionally recognised area with self-governing authority.^11^ Iraq is a country vulnerable to climate-related stressors: it is the fifth most vulnerable to climate change globally, with extreme temperatures, recurring droughts and increasing desertification accelerating its path towards uninhabitability in the coming decades.^12^ The country’s average temperature has increased by 0.5°C per decade since the 1970s, and summer temperatures regularly exceed 50°C.^13^ Climate variations across the country are influenced by latitude and high mountain ranges in the north. The northern mountainous regions receive higher rainfall (400–1000 mm per year) and colder temperatures, with snowfall in some areas during winter. In contrast, southern Iraq has a warm desert climate with significantly lower rainfall.^13^

In a study conducted in camps for internally displaced people in Ninewa and Duhok, 74% of respondents reported a direct impact of climate change on their daily lives, with extreme heat leading to increased aggression, anxiety and sleep disturbances.^14^ In the Kurdistan Region, research highlights that 19% of residents experience severe eco-anxiety, with urban populations showing higher levels of distress due to worsening environmental conditions.^15^

## The current study

The aim of this study is to examine the prevalence and impact of climate anxiety in Iraq, including the Kurdistan Region. It also seeks to analyse how demographic factors such as age, gender and geographical location influence climate anxiety levels. In addition, the study explores the relationship between climate anxiety and mental health, focusing on depression and generalised anxiety disorder, providing insight into its broader psychological effects.

## Method

### Study design and procedure

We used a cross-sectional study design that followed Strengthening the Reporting of Observational Studies in Epidemiology (STROBE) guidelines.^16^ To ensure the quality of the web-based survey, we also followed the Checklist for Reporting Results of Internet E-Surveys (CHERRIES).^17^ A convenience sampling technique was used to collect data. The online survey was distributed through social media platforms, including Facebook, LinkedIn, Instagram and WhatsApp. Before beginning the survey, general instructions including research information were provided, and an informed consent form was included in the first section of the questionnaire. Data collection was carried out between 23 January and 15 February 2025.

### Participants

A total of 1019 valid responses were received across 18 provinces. The inclusion criteria for participation in the study were: (a) being of Iraqi nationality, (b) residing within the geographical boundaries of Iraq at the time of data collection and (c) being 18 years of age or older. The exclusion criteria included (a) individuals under 18 years of age, (b) non-Iraqi nationals residing in Iraq and (c) Iraqi citizens living abroad.

### Measures

The first part of the questionnaires assessed the sociodemographic characteristics of the participants, including gender (male/female), age, occupation (student/private sector worker/government employee/retired/unemployed), marital status (single/married/divorced/widowed/), economic status (very poor/poor/middle income/rich), health status, in answer to the question ‘How do you describe your health in general?’ with options (very good/good/bad/very bad), and educational level (elementary/secondary/university).

In the second part of the questionnaire, participants completed three measures: a validated Arabic version of the Climate Change Anxiety Scale (CCAS),^18^ together with the Patient Health Questionnaire-9 (PHQ-9) and the Generalised Anxiety Disorder-7 questionnaire (GAD-7); the PHQ-9 and GAD-7 had been translated into Kurdish and Arabic and then back-translated into English by bilingual professionals to ensure accuracy.

#### Climate Change Anxiety Scale (CCAS)

The CCAS is a 13-item questionnaire designed to measure the degree of climate-related anxiety among individuals. Developed by Clayton & Karazsia (2020),^19^ it consists of two subscales: the cognitive impairment subscale (eight items; for example ‘Thinking about climate change makes it difficult for me to sleep’) and the functional impairment subscale (five items; for example ‘I have problems balancing my concerns about sustainability with the needs of my family’). Respondents evaluate how often the CCAS statements applied to them using a 5-point Likert scale, ranging from 1 (‘never’) to 5 (‘almost always’). The cut-off scores are as follows: scores <21 indicate no or minimal climate anxiety, scores between 21 and 22 indicate mild to moderate climate anxiety, and scores ≥23 indicate severe to extremely severe climate anxiety.^20^ The scale demonstrated good reliability (Cronbach’s *α* = 0.89).

#### Patient Health Questionnaire-9 (PHQ-9)

The PHQ-9 is a 9-item self-report measure that evaluates the severity of depressive symptoms over the previous 2 weeks.^21^ An example item includes ‘Thoughts that you would be better off dead or hurting yourself’. Responses are rated on a 4-point scale, from 0 (‘not at all’) to 3 (‘nearly every day’). Total scores range from 0 to 27, with 15–19 indicating moderately severe depression and ≥20 indicating severe depression. In this study, the PHQ-9 demonstrated good reliability (Cronbach’s *α* = 0.88).

#### Generalised Anxiety Disorder-7 (GAD-7)

The GAD-7 is a 7-item self-report tool designed to evaluate general anxiety levels.^22^ Each item is scored on a 4-point scale, ranging from 0 (‘not at all’) to 3 (‘nearly every day’), with total scores ranging from 0 to 21. A score ≥15 indicates severe generalised anxiety disorder. The GAD-7 demonstrated excellent reliability (Cronbach’s *α* = 0.91).

### Statistical analysis

The Statistical Package for the Social Sciences (SPSS) version 27.0 for Windows (IBM Corp., Armonk, NY, USA; see https://www.ibm.com/products/spss-statistics) was used for statistical analysis. Jupyter Notebook version 6.5.4, 2023 for Windows (Project Jupyter, open-source, see https://jupyter.org/) was used to generate regression graphs. ArcGIS Desktop Version 10.8 for Windows (Esri, Redlands, CA, USA; see https://www.esri.com/) was used to visualise the regional distribution of climate anxiety on a map. Cronbach’s *α* was calculated to assess the internal consistency of the study instruments. Descriptive statistics were used to analyse the participants’ sociodemographic characteristics and to determine the overall prevalence of climate anxiety. Independent-sample *t*-tests were performed to assess gender and age differences in CCAS scores, and one-way analysis of variance (ANOVA) was used to examine regional variations across different provinces. To estimate the impact of climate anxiety, we assessed its association with two key mental health outcomes: depression (PHQ-9) and generalised anxiety disorder (GAD-7). Pearson’s correlation analyses were performed to determine the associations between climate anxiety and mental health outcomes. Additionally, simple linear regression models were used to examine the predictive effect of climate anxiety on both depression and general anxiety levels. The strength of these models, represented by beta coefficients and *R* ^2^-values, provided a quantitative estimate of the psychological impact of climate anxiety on the study population.

## Results

### Participants

Table 1 shows sociodemographic characteristics of the participants. In total, 1019 participants were included in the study, with a slight majority being women (women: *n* = 532, 52.2%; men: *n* = 487, 47.8%). The mean age was 31.91 years (s.d. = 10.01, range 18–72 years) and the age was classified into two groups: young adults (18–41 years) and older adults (42–71 years). Most participants were in employment (58.4%) and classified themselves as middle income (88.8%). Just over half (50.5%) were married. Most reported good health (63.6%) and 92.0% had obtained higher education. The majority of the sample (75.6%) lived in the north of the country, in the Kurdistan Region of Iraq (KRI).

**Table 1:** Demographic characteristics of the participants (*n* = 1019) Table 1 long description.

Variables	Sub-variables	Frequency, *n*	Percentage
Gender	Male	487	47.8
	Female	532	52.2
Age	18–41 years (young adults)	854	83.8
	42–72 years (older adults)	165	16.2
Residency	Iraqi cities (central and southern Iraq)	249	24.4
	Kurdistan Region of Iraq (KRI)	770	75.6
Occupation	Student	283	27.8
	Private sector	183	18
	Government employee	409	40.1
	Retired	12	1.2
	Unemployed	132	13
Marital status	Single	474	46.5
	Married	515	50.5
	Separated	20	2
	Widowed	10	1
Economic status	Very poor	18	1.8
	Poor	56	5.5
	Middle income	905	88.8
	Rich	40	3.9
Health status (‘How do you describe your health in general?’)	Very good	311	30.5
	Good	648	63.6
	Bad	51	5
	Very bad	9	0.9
Educational level	Elementary	18	1.8
	Secondary	64	6.3
	University	937	92

### Prevalence and regional distribution of climate anxiety

The impact of climate change was observed across Iraq, and most (71.4%) participants reported severe levels of climate anxiety; only 21.8% reported minimal or no climate anxiety (Table 2). By region, more participants reported severe climate anxiety in the north (KRI, 73.2%) than in the central and southern provinces (65.9%). To explore the significance of climate anxiety in individual provinces in Iraq, we performed a sub-analysis in which provinces with a small sample size and no significant results on climate anxiety were removed. The results indicated that five provinces exhibited significantly higher levels of climate anxiety compared with others: Ninawa (mean 34.44, s.d. = 7.9), Basrah (mean 32.54, s.d. = 12.2), Najaf (mean 30.82, s.d. = 10.89), Duhok (mean 30.42, s.d. = 10.12) and Erbil (mean 30.22, s.d. = 9.58). One-way ANOVA identified significant differences in climate anxiety levels between some provinces (*F*(17,1001) = 2.92, *P* = 0.001, *η* ^2^ = 0.04). Figure 1 presents a map highlighting individual provinces with highest levels of climate anxiety.

**Table 2:** Prevalence of climate anxiety Table 2 long description.

Climate anxiety	Iraq in general		Central and southern provinces of Iraq		Kurdistan Region of Iraq	
	*n*	%	*n*	%	*n*	%
None/minimal	222	21.8	67	26.9	155	20.1
Mild/moderate	69	6.8	18	7.2	51	6.6
Severe/extremely severe	728	71.4	164	65.9	564	73.2

**Fig. 1:**
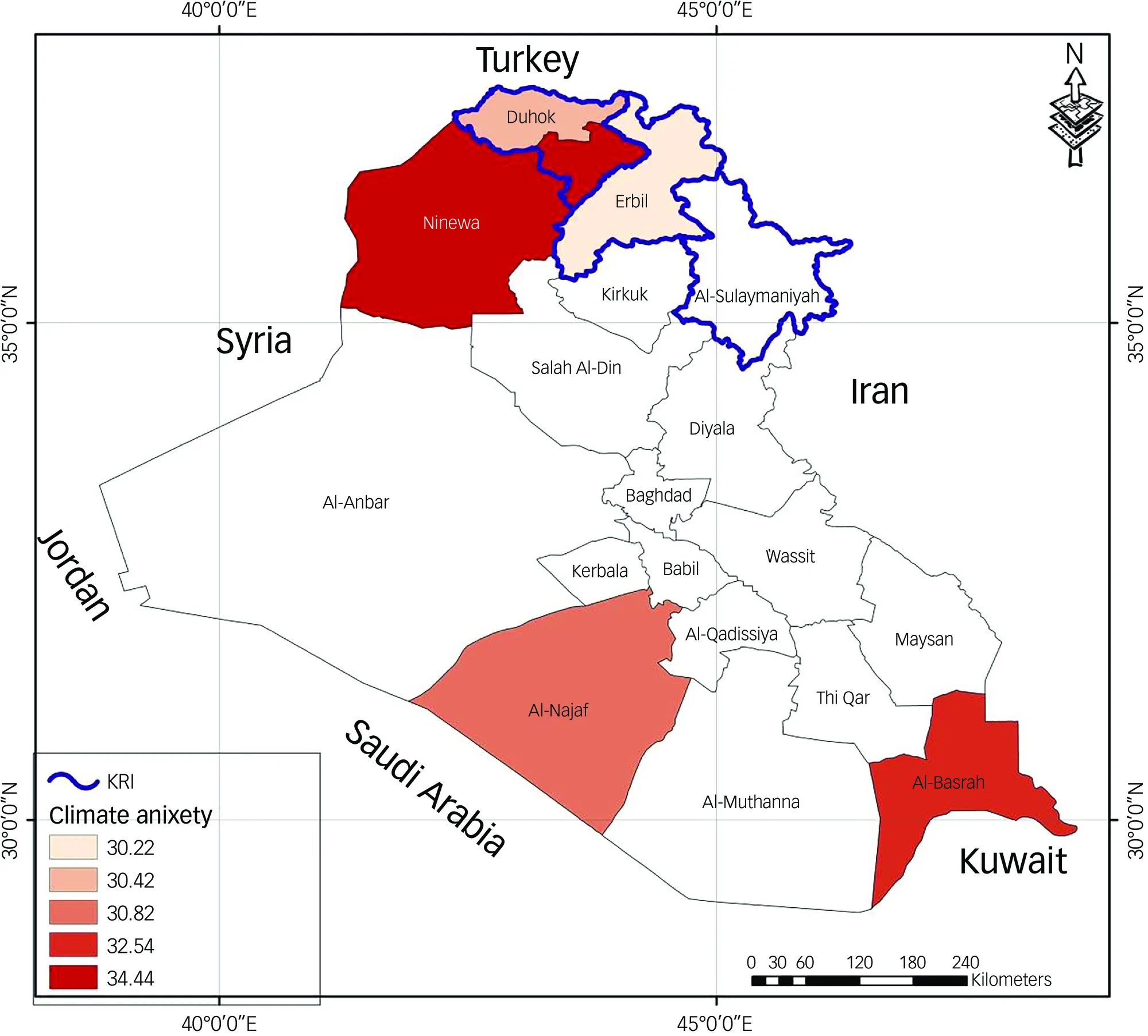
Iraqi provinces where participants experienced the highest levels of climate anxiety. KRI, Kurdistan Region of Iraq.

### Gender and age differences in climate anxiety

An independent-sample *t*-test revealed no significant differences in climate anxiety between males and females (Table 3). However, older adults (42–72 years) reported significantly higher climate anxiety than younger adults (18–41 years) (Cohen’s *d* = 0.22, indicating a small effect size).

**Table 3:** Gender and age differences in climate anxiety Table 3 long description.

Variable	*n*	Mean	s.d.	*t*	d.f.	Significance (2-tailed)	Point estimate, Cohen’s *d*
Gender							
Male	487	29.56	9.63	1.02	1017	0.306	–
Female	532	28.94	9.7				
Age group							
18–41 years	854	28.89	9.72	2.17	1017	0.008	0.22
42–72 years	165	31.06	9.21				

### Correlation and regression between climate anxiety and mental health (depression and generalised anxiety disorder)

A Pearson correlation analysis (Table 4) revealed a positive correlation between climate anxiety and depression and generalised anxiety disorder, indicating that higher climate anxiety was associated with increased symptoms of depression and anxiety.

**Table 4:** Correlation between climate anxiety, depression and generalised anxiety Table 4 long description.

Variable	Depression (PHQ-9)	Anxiety (GAD-7)
Climate anxiety	0.382**	0.361**
Significance	*P* < 0.001	*P* < 0.001

PHQ-9, Patient Health Questionnaire-9; GAD-7, Generalised Anxiety Disorder-7 questionnaire.

**The correlation is significant at the 0.01 level (2-tailed).

Furthermore, simple linear regression was significant (*β* = 0.25, *t* = 13.19, *P* < 0.001, 95% CI 0.219–0.206); the regression equation *F*(1,1017) = 174.3, *P* < 0.01, with *R* ^2^ = 0.146, indicated that climate anxiety represented 14.6% of the variance in depression, and the result was significant (*β* = 0.214, *t* = 12.32, *P* < 0.01, 95% CI 0.18– 0.248; the regression *F*(1,1017) = 151.99, *P* < 0.01, with *R* ^2^ = 0.13, indicated that climate anxiety accounted for 13% of the variance in generalised anxiety disorder. The regression results are shown in Fig. 2.

**Fig. 2:**
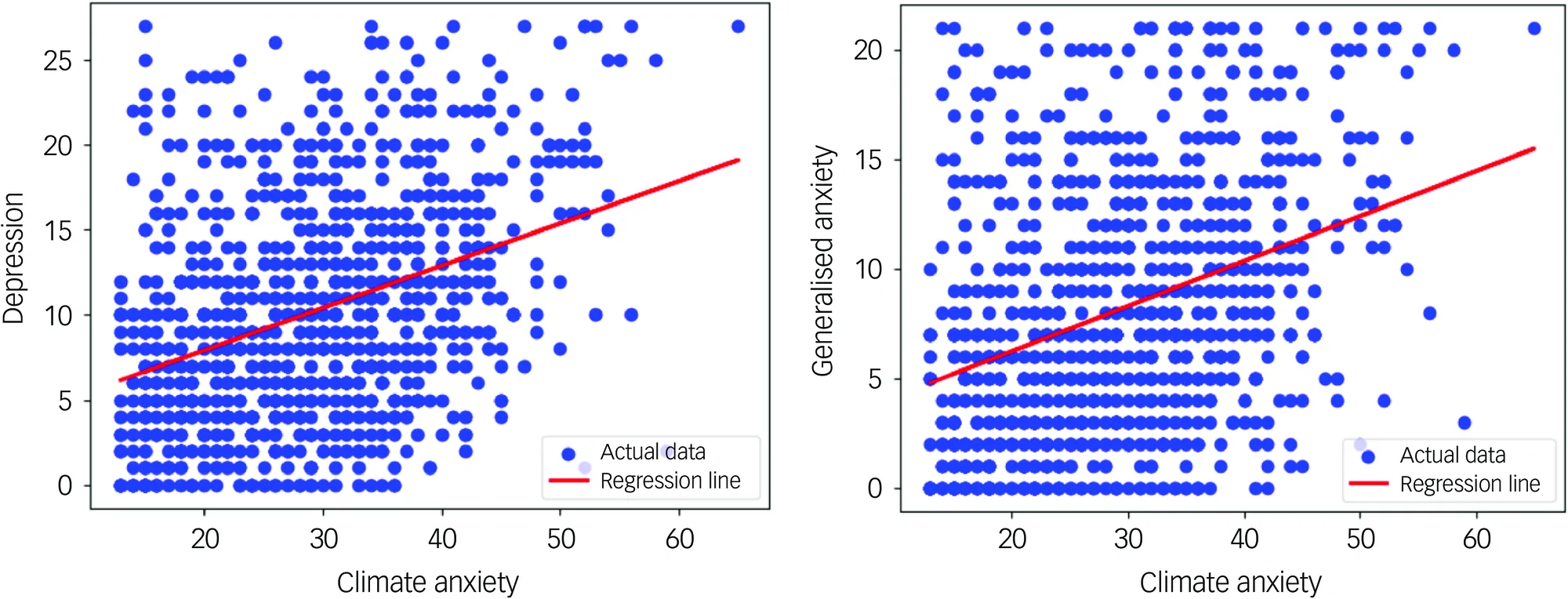
Regression model for climate anxiety, depression and generalised anxiety disorder.

## Discussion

The current study aimed to assess the prevalence and impact of climate anxiety and its association with mental health problems in Iraq. The findings revealed a substantial burden, with 71.4% of participants reporting severe levels of climate anxiety. In particular, severe climate anxiety was more prevalent in the Kurdistan Region (73.2%) compared with the central and southern Iraqi provinces (65.9%), and in the individual provinces of Ninawa, Basrah, Najaf, Duhok and Erbil.

Globally, studies have shown widespread anxiety related to climate change. Approximately 59% of people reported feeling very or extremely concerned, while 84% expressed at least moderate concern. Many also reported experiencing negative emotions such as sadness, anxiety, anger, powerlessness, helplessness, and guilt, which were associated with poorer mental well-being.^23–25^ Our study identified notable sociodemographic variations in climate anxiety. Age appeared to be a significant factor, with older adults reporting higher levels of climate anxiety than younger participants. This finding is consistent with other research that indicated that older individuals are more susceptible to climate anxiety, possibly because of their prolonged exposure to environmental changes and greater awareness of long-term consequences.^26^ On the contrary, the gender differences in climate anxiety in our study were not statistically significant, suggesting that men and women in Iraq experience similar levels of climate anxiety. However, this finding differs from research conducted among French-speaking people in eight European and African countries, where women were found to have significantly higher levels of climate anxiety than men.^27^ These variations suggest that sociocultural and environmental factors may influence the extent to which different demographic groups experience climate-related distress.

Regarding the association between climate anxiety and mental health problems, our study found a positive correlation in which higher levels of climate anxiety were significantly associated with increased depression and generalised anxiety disorder. Furthermore, while the regression analyses demonstrated a significant association between climate anxiety and both depression and generalised anxiety disorder, it is important to note that climate anxiety accounted for only 14.6% and 13% of the variance in depression and anxiety, respectively. This suggests that although climate anxiety contributes meaningfully to these mental health outcomes, it explains only a modest portion of the variance. Other unmeasured psychosocial, environmental or individual-level factors are likely to contribute substantially to symptoms of depression and generalised anxiety. These findings should caution readers against overestimating the clinical importance of climate anxiety in isolation and underscore the need for multifactorial models. Nevertheless, our results are consistent with global research indicating that climate anxiety is positively correlated with depression and anxiety disorders, particularly among vulnerable populations with limited access to mental health support, such as older adults and rural residents.^25,28–30^

### Recommendations and implications

Given Iraq’s ranking as one of the countries most vulnerable to climate change, the findings of this study emphasise that climate change should be recognised not only as an environmental crisis, but also as a pressing mental health challenge. It is essential that Iraq integrates mental health and psychosocial support (MHPSS) components into its national climate adaptation and disaster risk reduction strategies. The results demonstrate the need for targeted MHPSS interventions, particularly for older adults, individuals with pre-existing mental health conditions, and populations living in regions most severely affected by climate anxiety and environmental degradation. Future research should investigate the long-term psychological impacts of climate-related events, identify protective factors and assess the effectiveness of various psychosocial and community resilience-building strategies. Longitudinal and interdisciplinary studies are needed to explore how different demographic groups, such as youth, women and internally displaced persons, experience and respond to climate-related stressors. Furthermore, research should examine how factors such as social support, religious coping and traditional ecological knowledge influence mental health outcomes in the face of climate change.

Without proactive and coordinated action, the mental health consequences of climate change are likely to intensify, placing even greater strain on Iraq’s already fragile healthcare and social support systems. Addressing this emerging crisis requires collaboration between environmental policymakers, health professionals, humanitarian actors and researchers to ensure holistic, sustainable and inclusive responses.

### Strengths and limitations of the study

This study is among the first empirical investigations to explore climate anxiety and its mental health impacts across Iraq. However, several limitations should be disclosed. First, the use of a cross-sectional design prevents us from establishing causal relationships between climate anxiety and mental health outcomes. Second, the study relied on self-reported measures, which are subject to social desirability bias and potential recall inaccuracies. Furthermore, the sample was obtained through online recruitment; people in rural areas may be more affected by climate change; however, owing to illiteracy and limited knowledge of online platforms, their participation in the survey might be lacking. Furthermore, the overrepresentation of participants from the Kurdistan Region may have influenced the observed prevalence rates and regional comparisons. Future research should aim for a more balanced regional distribution and incorporate mixed-method approaches, including qualitative assessments, to gain a deeper understanding of the psychological burden of climate anxiety in Iraq.

## Table 1 Long description

The table presents sociodemographic characteristics of 1019 participants. It has 8 rows and 4 columns. The columns are labeled Variables, Sub-variables, Frequency, n, and Percentage. The rows are labeled with different variables and their sub-variables. Row 1: Gender, Male, 487, 47.8. Row 2: Gender, Female, 532, 52.2. Row 3: Age, 18-41 years (young adults), 854, 83.8. Row 4: Age, 42-72 years (older adults), 165, 16.2. Row 5: Residency, Iraqi cities (central and southern Iraq), 249, 24.4. Row 6: Residency, Kurdistan Region of Iraq (KRI), 770, 75.6. Row 7: Occupation, Student, 283, 27.8. Row 8: Occupation, Private sector, 183, 18. Row 9: Occupation, Government employee, 409, 40.1. Row 10: Occupation, Retired, 12, 1.2. Row 11: Occupation, Unemployed, 132, 13. Row 12: Marital status, Single, 474, 46.5. Row 13: Marital status, Married, 515, 50.5. Row 14: Marital status, Separated, 20, 2. Row 15: Marital status, Widowed, 10, 1. Row 16: Economic status, Very poor, 18, 1.8. Row 17: Economic status, Poor, 56, 5.5. Row 18: Economic status, Middle income, 905, 88.8. Row 19: Economic status, Rich, 40, 3.9. Row 20: Health status (How do you describe your health in general?), Very good, 311, 30.5. Row 21: Health status (How do you describe your health in general?), Good, 648, 63.6. Row 22: Health status (How do you describe your health in general?), Bad, 51, 5. Row 23: Health status (How do you describe your health in general?), Very bad, 9, 0.9. Row 24: Educational level, Elementary, 18, 1.8. Row 25: Educational level, Secondary, 64, 6.3. Row 26: Educational level, University, 937, 92.

Navigate back to Table 1.

## Table 2 Long description

The table presents the prevalence of climate anxiety across Iraq, divided into three regions: Iraq in general, Central and southern provinces of Iraq, and Kurdistan Region of Iraq. It has four rows and three columns. The columns are labeled 'Iraq in general', 'Central and southern provinces of Iraq', and 'Kurdistan Region of Iraq'. The rows are labeled 'Climate anxiety' with subcategories 'None/minimal', 'Mild/moderate', and 'Severe/extremely severe'. Each cell contains values for 'n' and '%'. Row 1: None/minimal, Iraq in general, n: 222, percent: 21.8; Central and southern provinces of Iraq, n: 67, percent: 26.9; Kurdistan Region of Iraq, n: 155, percent: 20.1. Row 2: Mild/moderate, Iraq in general, n: 69, percent: 6.8; Central and southern provinces of Iraq, n: 18, percent: 7.2; Kurdistan Region of Iraq, n: 51, percent: 6.6. Row 3: Severe/extremely severe, Iraq in general, n: 728, percent: 71.4; Central and southern provinces of Iraq, n: 164, percent: 65.9; Kurdistan Region of Iraq, n: 564, percent: 73.2.

Navigate back to Table 2.

## Table 3 Long description

The table presents the results of an independent-sample t-test analyzing climate anxiety differences based on gender and age groups. It includes two main categories: Gender and Age group, each with corresponding rows for specific groups. The columns are labeled as Variable, n, Mean, s.d., t, d.f., Significance (2-tailed), and Point estimate, Cohen's d. For Gender, the table shows data for Male and Female groups with sample sizes of 487 and 532, respectively. The Mean climate anxiety scores are 29.56 for males and 28.94 for females, with standard deviations of 9.63 and 9.7, respectively. The t-value is 1.02 with 1017 degrees of freedom and a significance level of 0.306. For Age group, the table shows data for 18-41 years and 42-72 years with sample sizes of 854 and 165, respectively. The Mean climate anxiety scores are 28.89 for the younger age group and 31.06 for the older age group, with standard deviations of 9.72 and 9.21, respectively. The t-value is 2.17 with 1017 degrees of freedom and a significance level of 0.008. The Point estimate, Cohen's d, for the age group comparison is 0.22, indicating a small effect size.

Navigate back to Table 3.

## Table 4 Long description

A table with two rows and three columns. The columns are labeled 'Variable', 'Depression (PHQ-9)', and 'Anxiety (GAD-7)'. The rows are labeled 'Climate anxiety' and 'Significance'. Row 1: Climate anxiety, 0.382**, 0.361**. Row 2: Significance, P < 0.001, P < 0.001. The table indicates a positive correlation between climate anxiety and both depression and anxiety, with significance levels noted.

Navigate back to Table 4.
